# Opportunities for Quantum Sensing in Epilepsy Diagnostics

**DOI:** 10.3390/s26175490

**Published:** 2026-08-29

**Authors:** Dina Kalinina, Alexandr Pak, Mukhit Dossov, Zhassulan Utebekov, Gaziz Kyrgyzbay, Darkhan Kimadiev, Guldana Zhumabayeva, Francisco Ulises Hernandez Ledezma, Jose Berengueres

**Affiliations:** 1 Biological and Biomedical Sciences Department, School of Medicine, Nazarbayev University, Astana Z05P3Y4, Kazakhstan; apak@nu.edu.kz; 2 Anesthesiology & ICU Department, RSE Medical Centre Hospital of the President’s Affairs Administration of the Republic of Kazakhstan, Astana Z05M4E8, Kazakhstan; dossovmukhit@gmail.com; 3 Epileptology Centre, RSE Medical Centre Hospital of the President’s Affairs Administration of the Republic of Kazakhstan, Astana Z05M4E8, Kazakhstan; jase8522@gmail.com (Z.U.); doctorgaziz@gmail.com (G.K.); kimadiev96@gmail.com (D.K.); dana922012@gmail.com (G.Z.); 4 Skydiamond Quantum, Lion House, Rowcroft, Stroud, Gloucestershire GL5 3BY, UK; ulises.hernandez@skydiamond.com; 5 Computer Science Department, School of Computing and AI, Nazarbayev University, Astana Z05H0P9, Kazakhstan

**Keywords:** quantum sensing, epilepsy, presurgical evaluation, magnetoencephalography, neurophysiology

## Abstract

**Highlights:**

**What are the main findings?**

- OPM-MEG has progressed from proof-of-concept in 2020 to small-cohort clinical feasibility studies, demonstrating IED detection and source localisation comparable to SQUID-MEG, with early evidence of benefit in paediatric populations and mesial temporal lobe epilepsy.
- Solid-state quantum sensors (nitrogen-vacancy diamond and silicon carbide divacancy) remain preclinical, constrained by a fundamental standoff-sensitivity trade-off, though recent advances—including sub-10 pT/√Hz nitrogen-vacancy diamond sensitivity at 1 mm standoff—indicate that near-field feasibility is an active area of progress.

**What are the implications of the main findings?**

- OPM-MEG is best positioned as a complementary modality within presurgical evaluation workflows; its primary value derives from geometric (reduced standoff) rather than sensitivity advantages over SQUID-MEG, and prospective multicentre outcome studies are the critical next step toward guideline-level evidence.
- The convergence of quantum sensing with quantum computing, machine learning, and genetically encodable biosensors outlines a longer-term trajectory that may address unresolved challenges in the source localisation of epileptiform activity and high-resolution near-field neural sensing.

**Abstract:**

Diagnosis in epilepsy depends on several distinct objectives—including interictal epileptiform discharge (IED) detection, source localisation of epileptiform activity, and seizure classification—each shaped by the underlying sensing technology. This review examines the opportunities that quantum magnetic sensing offers within this diagnostic framework, with particular emphasis on translational readiness and clinical potential. Seizure classification is included to situate the broader clinical context of epilepsy diagnostics, whereas the review of quantum sensing evidence is focused on IED detection and presurgical source localisation, the objectives for which such evidence currently exists. Magnetoencephalography (MEG) provides non-invasive source localisation that complements electroencephalography (EEG), and its clinical adoption is increasingly determined by advances in magnetic sensor technology. Optically pumped magnetometer (OPM)-based MEG represents the most clinically developed quantum sensing application, progressing from proof-of-concept in 2020 toward small-cohort clinical feasibility studies from 2022 onwards. By enabling on-scalp recordings at reduced sensor-to-brain distance, OPM-MEG improves signal amplitude and permits flexible sensor placement, with early evidence of benefit in paediatric populations and mesial temporal lobe epilepsy. However, current evidence derives from small, heterogeneous cohorts, and no prospective data yet link OPM-MEG to surgical outcomes. ^4^He-OPMs represent an early feasibility stage with IED detection comparable to superconducting quantum interference device (SQUID)-MEG in small case series; nitrogen-vacancy centres in diamond and silicon carbide divacancy magnetometry remain preclinical, constrained by a fundamental standoff-sensitivity trade-off. We conclude that OPM-MEG is best positioned as a complementary modality within established presurgical workflows, with prospective multicentre outcome studies representing the critical next step toward clinical translation.

## 1. Introduction

Epilepsy is a chronic neurological condition clinically defined by either two unprovoked seizures occurring more than 24 h apart or one unprovoked seizure when the probability of further seizures is sufficiently high [1]. In 2017, International League Against Epilepsy (ILAE) classified seizures into the following three groups according to the onset type: focal, generalised, or unknown, and further subclassified them by semiology, including motor and non-motor features as well as level of awareness in focal seizures [2,3].

**Diagnostic objectives and terminology.** Accurate epilepsy diagnosis rests on several distinct objectives that are often conflated but require different sensing capabilities. It is useful to distinguish them at the outset. Ictal seizure detection identifies when a seizure occurs in real time and is the domain of ambulatory monitoring. Interictal epileptiform discharge (IED) detection identifies epileptiform activity between seizures and is central to presurgical evaluation because IEDs are far more frequent than seizures themselves. A further distinction separates sensor-level detection, meaning recognition that abnormal activity is present, from source localisation, the reconstruction of the cortical origin of that activity through forward and inverse modelling. Localisation itself is not a single target: the irritative zone (the region generating IEDs) is not necessarily equivalent to the epileptogenic zone (the region whose removal is required for seizure freedom), and both differ from the seizure-onset zone observed during ictal recordings. Finally, the following two further objectives carry their own evidentiary standards: seizure classification (determining seizure type) and clinical outcome prediction (forecasting surgical success). Throughout this review we therefore use source localisation to mean the reconstruction of the cortical generators of recorded epileptiform activity, which corresponds most closely to the irritative zone, and we reserve the term epileptogenic zone for the multimodally inferred target of resection, which is confirmed only retrospectively by post-surgical seizure freedom. MEG and OPM-MEG are accordingly described as localising epileptiform activity that contributes evidence towards delineation of the epileptogenic zone, rather than as determining the epileptogenic zone directly. These objectives also differ in the extent to which quantum sensing has been applied to them. Ictal seizure detection and seizure classification are described here to establish the clinical context in which magnetic sensing would be used, whereas the quantum sensing literature reviewed in Section 3 and Section 4 addresses IED detection and presurgical source localisation, and the scope of this review is delimited accordingly.

### 1.1. Diagnostic Challenges

Sensor-based epilepsy diagnosis dates to the 1930s, when EEG entered clinical use. Advances in sensing capability have been a principal driver of progress across these diagnostic objectives. The current clinical challenges within each are outlined below.

**Detection**. Approximately half of seizures go undetected by patients [4,5], with false negatives carrying direct clinical consequences including elevated risk of sudden unexpected death in epilepsy (SUDEP), which accounts for 8–17% of all epilepsy-related deaths [6]. EEG has traditionally been the primary detection tool, and its analysis has evolved considerably as follows: from expert visual interpretation of EEG established in the 1930s to automated signal-processing approaches and, more recently, machine learning and deep learning models [7]. One drawback of EEG is that it requires fixed infrastructure, restricting monitoring to clinical settings. Wearable non-EEG devices have emerged to address ambulatory detection needs, but their performance for focal and non-convulsive seizures remains substantially limited by several factors [8,9]. Alongside, patient seizure diaries remain widely used in clinical practice. Unfortunately, they have been shown to be systematically inaccurate. Approximately half of seizures captured on concurrent video-EEG go unreported [4,5].

**Localisation.** In approximately 30% of patients with epilepsy, seizures remain refractory to medication, and surgical resection becomes a treatment option [10]. In such cases, correct delineation of the epileptogenic zone is critical to surgical success. Because the epileptogenic zone is a conceptual construct that no single modality measures directly, it is inferred from the convergence of several localising findings, each of which identifies a related but distinct cortical zone. This requires high spatial precision within a multimodal diagnostic framework [11,12].

**Classification.** EEG remains the primary classification tool, as it offers a direct measurement of the cortical electrical activity across a wide range of seizure types and settings. To improve the odds of correct classification, EEG is increasingly combined with magnetoencephalography (MEG), particularly in the evaluation of drug-resistant focal epilepsy.

### 1.2. Quantum Sensing in Epilepsy

A key determinant of surgical outcome is source localisation accuracy [13,14]. MEG measures magnetic fields generated primarily by synchronised postsynaptic intracellular currents in cortical pyramidal neurons, whereas EEG measures the resulting extracellular potential differences at the scalp [15,16]. Then, through forward and inverse modelling, the cortical generators of the recorded epileptiform activity can be estimated [15,17]. These source estimates delineate the irritative zone and contribute evidence towards, but do not by themselves determine, the epileptogenic zone [11,12]. In this, one advantage of MEG over EEG is that unlike electric potentials, magnetic fields are not as distorted by the differential properties (mainly conductivity) of brain tissue, cerebrospinal fluid, skull, and scalp. In a roughly spherical volume conductor, MEG is most sensitive to tangential cortical sources, such as those in sulcal walls, and less sensitive to radially oriented sources on gyral crowns. As mentioned, combining EEG with MEG has been shown to improve accuracy [18].

Clinically deployed MEG systems rely on quantum-limited magnetic sensors, historically SQUIDs and increasingly OPMs. The following two generations of such systems exist: those based on superconducting quantum interference devices (SQUIDs) and those based on optically pumped magnetometers (OPMs)—first available in the 2010s. Traditional cryogenic SQUID-based systems require liquid helium cooling and rigid, fixed installations. In contrast, room-temperature OPM enables direct on-scalp placement, flexible helmet designs, and recordings during natural head movement [19]. First demonstrated in an epilepsy patient in 2020 [20], OPM-MEG has since become the most clinically developed quantum sensing modality in epilepsy, with cohort studies from 2022 onwards demonstrating IED detection and source localisation comparable to SQUID-MEG [21,22,23].

**Solid-state room-temperature quantum sensors**. Distinct from vapour-cell OPM systems described above, solid-state room-temperature quantum sensors include nitrogen-vacancy (NV) centres in diamond and silicon carbide (SiC) divacancy qubits. Both are in early-stage development, with potential advantages for near-tissue and implantable sensing that are later examined. Neither NV-diamond nor SiC-divacancy magnetometry has yet demonstrated clinically useful human neuromagnetic recording for epilepsy [24,25]. However, because of their lower cost, solid-state and smaller size, they have potential to complement cryogenic-based devices (e.g., invasive or near-tissue sensing). To better understand the current situation, this review first traces the technological evolution of seizure detection and localisation, from classical electrophysiology to modern multimodal systems, and it then focuses on how room-temperature sensors may address current limitations. In the Section 4 it reviews how these new sensor tools might shape future clinical practice in epilepsy.

## 2. Materials and Methods

This narrative review examines the translational readiness and clinical potential of quantum sensing modalities in epilepsy diagnostics, including vapour-cell OPM systems and solid-state room-temperature quantum sensors. Although epilepsy diagnostics also encompasses ictal seizure detection and seizure classification, which are outlined in Section 1 to provide clinical context, the appraisal of quantum sensing evidence presented here is restricted to IED detection and presurgical source localisation; no quantum sensing study identified in our searches addressed seizure classification as a primary objective, and no claims regarding classification performance are therefore made.

Literature searches were conducted in PubMed, Embase, and Google Scholar, supplemented by IEEE Xplore for instrumentation and the engineering literature relevant to quantum sensing. No publication-date restrictions were applied; the final search update was performed in June 2026. The exact calendar day was not prospectively recorded. Searches used combinations of the terms (epilepsy OR seizure) AND (magnetoencephalography OR MEG OR optically pumped magnetometer OR OPM OR SQUID OR quantum sensing OR neuromagnetic OR nitrogen-vacancy OR NV-diamond OR silicon carbide OR SiC OR wearable seizure detection OR interictal epileptiform discharge). Records were included if they reported original data, methodological development, or quantitative synthesis relevant to seizure detection, IED detection, source localisation, or quantum sensing technologies applicable to epilepsy and were published in English. Conference abstracts without full-text data, commercial product materials, and the non-peer-reviewed grey literature were excluded, with the following two exceptions: two preprints were retained because their content was directly relevant to the review’s conclusions and not yet available in peer-reviewed form—Mellor et al. (2024) [26], for mesial temporal OPM-MEG recording, and Wesseler and Nagy (2026) [27], for a fibre-coupled NV-diamond sensitivity result—both explicitly labelled as preprints wherever cited. Preprints were treated as preliminary evidence and were not used alone to support claims of clinical readiness. Studies in healthy participants were included only where they characterised sensor performance directly transferable to epilepsy applications and are distinguished from clinical epilepsy studies in the text.

Consistent with a narrative rather than systematic review, no PRISMA protocol was applied, the number of records screened was not formally tracked, and screening and selection were performed by the first author; no independent second-reviewer cross-check was performed. Study quality was assessed qualitatively, with attention to sample size, study design (prospective, retrospective, or case series), validation approach (comparison with concurrent SQUID-MEG, intracranial EEG or SEEG, or surgical outcome data), and consistency of reporting including signal metrics, source localisation methods, and concordance measures. No formal risk-of-bias instrument was applied. These methodological choices, and the small sample sizes of the underlying clinical literature, are acknowledged as limitations.

## 3. Results

### 3.1. Current Diagnostic Methods in Epilepsy

#### 3.1.1. Electroencephalography

Clinical epilepsy diagnosis has rested on EEG since the 1930s, when Gibbs, Davis, and Lennox described the 3 Hz spike-and-wave discharge of absence seizures and Gibbs and Jasper characterised focal interictal spikes, establishing the IED as a lasting biomarker of epileptic activity [28,29]. EEG remains the primary tool for seizure classification and detection, offering direct measurement of cortical electrical activity across a wide range of seizure types. Its analysis has evolved from expert visual interpretation toward automated and, more recently, deep learning approaches [7]. However, these computational methods still face poor cross-patient generalisation arising from the non-stationarity of EEG and inter-individual variability in ictal morphology, elevated false-alarm rates from movement and physiological artefacts, and an absence of prospective validation against clinical endpoints; manual EEG review by trained clinicians accordingly remains the operational gold standard [7,30]. A further practical constraint is that scalp EEG requires fixed recording infrastructure and specialist interpretation, confining it largely to controlled clinical environments [31].

#### 3.1.2. Wearable Non-EEG Systems

The need for ambulatory monitoring has driven the development of wearable systems based on movement and autonomic physiology rather than direct cortical recording. Poh et al. first demonstrated detection of generalised tonic–clonic (GTC) seizures using a wrist-worn device combining electrodermal activity (EDA) and accelerometery, reporting 94% sensitivity at a false-alarm rate of 0.74 per 24 h across 31 patients who contributed 54 GTC seizures over more than 4200 h of monitoring [32]. Subsequent multimodal systems integrating accelerometery, EDA, photoplethysmography-derived heart rate, and surface electromyography have been validated in prospective multicentre studies and cleared for GTC monitoring in ambulatory settings [33,34]. Performance nevertheless remains strongly seizure-type dependent: a meta-analysis of 28 studies found an overall false-alarm rate of 2.1 per 24 h, with substantially lower sensitivity for focal seizures without bilateral spread than for convulsive events [8]. The underlying reason is neurophysiological—convulsive seizures produce widespread, time-locked motor, cardiovascular, and electrodermal changes that are reliably detectable peripherally, whereas focal seizures without prominent autonomic or motor components leave weak or inconsistent peripheral signatures [9].

**Figure 1:**
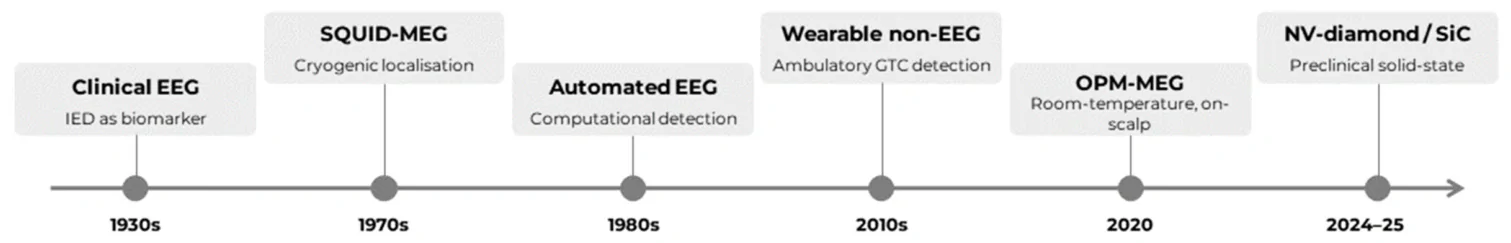
Evolution of sensing technologies in epilepsy diagnostics. Epilepsy sensing has evolved from clinical EEG through cryogenic SQUID-MEG to room-temperature OPM-MEG, the most clinically developed quantum sensing modality, with solid-state platforms (NV-diamond, SiC divacancy) representing emerging preclinical directions. EEG = electroencephalography; GTC = generalised tonic–clonic; IED = interictal epileptiform discharge; MEG = magnetoencephalography; OPM = optically pumped magnetometer; SQUID = superconducting quantum interference device; NV = nitrogen-vacancy; SiC = silicon carbide.

Thus, the technological landscape bifurcates as follows: peripheral wearables have advanced ambulatory detection of convulsive seizures but cannot localise their origin, whereas non-invasive source localisation of epileptiform activity, which is the focus of the remainder of this review, depends on magnetic sensing technologies (Figure 1).

### 3.2. Quantum Sensing for Epilepsy

#### 3.2.1. The Biophysical Basis of Magnetic Sensing in Epilepsy

Quantum sensing exploits quantum mechanical phenomena to measure physical quantities with sensitivity that approaches or surpasses the limits achievable by classical devices such as inductive coil-based magnetometers. In epilepsy, the relevant physical quantity is the extracranial magnetic field generated by synchronised postsynaptic currents in cortical pyramidal neurons, which is the same neuronal activity that underlies seizures and interictal discharges. These fields are extraordinarily weak, in the range of 10–100 femtotesla (fT), approximately five to six orders of magnitude smaller than the Earth’s static magnetic field and well below ambient urban electromagnetic noise, imposing stringent requirements on sensor sensitivity and environmental noise suppression [15,17]. SQUIDs and OPMs achieve this femtotesla-range sensitivity non-invasively—a level unattainable by classical coil-based sensors. In doing so, they measure the extracranial magnetic fields generated by neuronal currents with millisecond temporal resolution, enabling reconstruction of their cortical origin through source modelling, rather than relying on the secondary peripheral consequences of seizures (such as movement or autonomic change) that wearable systems detect.

#### 3.2.2. SQUID-Based MEG

Conventional helmet MEG systems rely on SQUIDs, which operate at cryogenic temperatures near absolute zero (approximately −269 °C) maintained by liquid helium dewars. The thermal insulation required to sustain this cooling necessitates a rigid, fixed-geometry helmet in which sensors sit approximately 20 mm from the scalp surface in adults, a distance that increases substantially in children due to size mismatch between their smaller heads and the one-size-fits-all adult helmet [14,35]. Commercial SQUID systems typically comprise 200–300 sensors and achieve noise floors in the low femtotesla-per-root-hertz range, enabling reliable detection of neuromagnetic signals in appropriately shielded environments [17]. In the clinical setting, SQUID-MEG is an established modality for presurgical evaluation of drug-resistant epilepsy. When MEG source localisation yields a focal generator of epileptiform activity that agrees with other presurgical findings, seizure freedom is approximately five times more likely following resection, and complete resection of MEG-identified IED clusters is independently associated with favourable surgical outcome [13,14]. The substantial infrastructure requirements restrict access to a small number of tertiary centres worldwide, and MEG remains outside the standard presurgical pathway in the majority of institutions [14,36].

#### 3.2.3. Optically Pumped Magnetometers MEG

OPMs detect magnetic fields through optical pumping of an alkali-metal vapour, most commonly rubidium or caesium, contained in a small heated glass cell. Circularly polarised laser light tuned to an atomic transition polarises the atomic spins, aligning them along the beam axis; an external magnetic field causes these spins to precess, altering the vapour’s optical transparency, which is read out as a change in transmitted light intensity [19,37]. Because the sensing element is an atomic vapour rather than a cryogenic superconductor, OPMs operate at or near room temperature and can be miniaturised to a few cubic centimetres, allowing them to be mounted directly on the scalp.

The highest sensitivity is achieved in the spin-exchange relaxation-free (SERF) regime, in which the vapour cell is heated to increase atomic density and operated in a very low ambient magnetic field. Under these conditions, the rate of spin-exchange collisions between atoms exceeds the spin precession frequency, suppressing a dominant relaxation mechanism and enabling femtotesla-level sensitivity. Contemporary commercial SERF-OPM sensors typically achieve noise floors in the range of 7–15 fT/√Hz, rather than the sub-femtotesla figures sometimes cited for optimised laboratory systems. SERF operation, however, requires that the background field at the sensor be reduced to near zero, since the regime collapses at fields above a few nanotesla. This imposes the following two practical constraints: alkali OPMs have a narrow dynamic range (approximately ±5 nT) and a limited bandwidth (roughly 1–100 Hz), and they require both a magnetically shielded room and active field compensation. Field-nulling coil arrays surrounding the participant cancel the residual static field and, critically, suppress the field changes generated when the head moves, without which even small head rotations would saturate the sensors. This active field control is what permits movement-tolerant recording and is a prerequisite for the flexible, participant-adapted helmet designs that distinguish OPM-MEG from fixed-geometry SQUID systems.

Because these sensors can be placed within a few millimetres of the scalp, rather than the approximately 20 mm standoff imposed by SQUID cryogenics, OPM-MEG captures neuromagnetic signals of substantially higher amplitude and improved signal-to-noise ratio (SNR), which is directly relevant to ictal recordings and to paediatric populations in whom sustained stillness cannot be maintained [19,35,38]. The first demonstration of OPM-MEG in an adult epilepsy patient with unrestricted head movement was reported in 2020 [20], and wearable OPM systems capable of recording IEDs in infants, children, and adults in movement-tolerant conditions within a magnetically shielded room have since been established [38].

Early clinical studies have established proof-of-concept and technical comparability with SQUID-MEG across paediatric and adult cohorts. In a prospective study of 14 school-aged children, on-scalp OPM-MEG detected IEDs with amplitudes 2.3–4.6 times higher and signal-to-noise ratios 27–60% greater than concurrent cryogenic SQUID-MEG, with source localisation concordance within approximately 5–15 mm [21]. A subsequent neurodevelopmental study in healthy participants aged 2–13 years, though not an epilepsy cohort, demonstrated that head-size-adaptable OPM helmets maintain consistent sensor proximity across age groups, establishing the feasibility of lifespan-wide paediatric recording that is directly relevant to paediatric epilepsy applications [39]. Comparative studies in adult cohorts have confirmed that OPM-MEG achieves IED detection rates and source localisation comparable to SQUID-MEG, with consistent amplitude and signal-to-noise advantages attributable to reduced sensor-to-brain distance [22,23]. OPM-MEG has additionally been compared with SEEG in patients undergoing presurgical invasive evaluation, with a single-centre case series demonstrating concordant detection of IEDs and epileptic network activity against simultaneous intracranial recording as ground truth [40]. Across these studies, however, sample sizes are small (*n* = 1–46), designs are predominantly single-centre case series with sequential rather than randomised comparison, and no study has yet linked OPM-MEG findings to surgical outcomes—so the current evidence supports technical feasibility and signal-level comparability rather than demonstrated clinical benefit.

Current alkali OPM sensors (typically rubidium-based) have a practical bandwidth of approximately 1–100 Hz and a dynamic range of roughly ±5 nT, requiring more demanding magnetic shielding than SQUID systems and generating sensor heat that must be managed to avoid patient discomfort at high sensor densities [40]. Helium-4 OPMs (^4^He-OPM) represent a complementary development operating at ambient temperature with a wider dynamic range (>200 nT) and bandwidth extending to approximately 2 kHz, theoretically enabling recording of high-frequency oscillations (ripples and fast ripples, 80–500 Hz) that carry localising value in presurgical evaluation [41,42]. In comparative studies, ^4^He-OPM systems have demonstrated IED detection with signal-to-noise ratio comparable to or higher than SQUID-MEG, attributed to closer sensor placement, despite a current intrinsic noise level of approximately 40–50 fT/√Hz, higher than the 3–5 fT/√Hz of state-of-the-art SQUID magnetometers [40,41]. Ongoing technical development is expected to reduce this noise gap; recent engineering advances have produced miniaturised, integrated OPM-MEG electronics with improved dynamic range through closed-loop sensor operation, reducing system complexity and improving deployability [43].

#### 3.2.4. Nitrogen-Vacancy Centre Magnetometry in Diamond

NV-diamond magnetometry uses optically detected magnetic resonance of nitrogen-vacancy centres to infer local magnetic fields [44]. It can work without saturating in fully ambient conditions and it is effective at nanometre-to-micrometre spatial scales, with broad potential applicability in life sciences owing to the biocompatibility of diamond [24,45,46]. The following two distinct application pathways exist: bulk diamond platforms, which support contact-based measurements such as NV-based nuclear magnetic resonance (NMR) and imaging of cells or tissue slices placed directly on the diamond surface, and nanodiamond approaches, in which fluorescent nanodiamonds containing NV centres are introduced intracellularly, enabling quantum sensing within living cells [45]. Proof-of-concept experiments have demonstrated detection of action potential magnetic fields from excised single neurons at sensor-to-sample distances of approximately 10 µm [24], and simulation studies have extended this framework to neural population activity in brain slices [47]. More recently, NV-based sensing has been applied to action potentials in live mammalian muscle tissue, demonstrating 50 pT/√Hz sensitivity under ambient, unshielded laboratory conditions, an important step toward biological applications, though as yet uncompetitive with probe electrophysiology in terms of sensitivity [48]. Diamond nanostructuring approaches, including photonic cavity integration and covariance magnetometry, offer a route to substantially improved sensitivity that may eventually close this gap [49]. Although NV sensors remain less sensitive than OPMs for macroscopic neuromagnetic fields, their small active sensing volume enables substantially higher spatial and temporal resolution at near-contact distances—achieving nanometre-to-micrometre spatial resolution and microsecond-to-millisecond temporal resolution that is unattainable by conventional EEG, MEG, or fMRI [50]. One drawback of NV-diamond is that NV centres require green light excitation (532 nm), which is strongly absorbed by organic tissue and water, causing tissue heating and autofluorescence—a physical constraint that limits in vivo applicability. Several approaches have been developed to mitigate these limitations, including multiphoton near-infrared excitation [51,52], upconversion-assisted excitation using near-infrared wavelengths [53], time-gated detection, and modulation-based background rejection to suppress autofluorescence in biological systems [54,55]. These strategies have not yet been demonstrated in intact neural tissue but represent active engineering directions that may extend the in vivo applicability of NV-diamond sensing. These constraints currently require intimate proximity between sensor and tissue, and at macroscopic distances NV-diamond remains insufficient for scalp-level neuromagnetic recording. Recent advances are nonetheless narrowing this gap as follows: in 2024, a continuous-wave optically detected magnetic resonance (ODMR) NV-diamond magnetometer achieved sub-10 pT/√Hz sensitivity (9.4 ± 0.1 pT/√Hz) in the clinically relevant 5–100 Hz range, at a measurement distance of approximately 1 mm and without a magnetic flux concentrator [56]. This represents a significant advance toward practical, ambient-condition magnetometry with millimetre-scale spatial resolution, though the standoff remains far shorter than scalp-level recording requires, and validated scalp-level epilepsy MEG has not been demonstrated. The field is therefore best characterised as an emerging technology for magnetometry in brain mapping and bioimaging rather than a clinically ready one [50].

#### 3.2.5. Silicon Carbide Divacancy

Silicon carbide divacancy qubits [25] offer two principal advantages over NV-diamond. First, as a silicon-based semiconductor material, SiC integrates readily with existing chip fabrication processes, including system-on-chip (SoC) architectures. Second, unlike NV, SiC divacancy qubits are excited and read out at near-infrared wavelengths that fall within the second biological window, where absorption is minimal. This makes them inherently more suitable for biological applications [25]. A recent variant uses alkene-terminated 4H-SiC surfaces hosting shallow divacancy qubits, which have been shown to maintain stable room-temperature operation with a bioinert host material and theoretical magnetometry sensitivity of approximately 13 nT/√Hz for a single divacancy sensor in isotope-engineered material [25]. Although SiC offers stronger wafer-scale integration during fabrication, both SiC divacancy and NV-diamond platforms have demonstrated feasibility for coupling with compact packaging solutions and optical fibre systems. A fibre-coupled SiC divacancy magnetometer with simultaneous thermometry has been demonstrated, achieving a sensitivity of 3.9 µT/√Hz [57]. A fibre-coupled NV bulk diamond magnetometer demonstrated a magnetic field sensitivity of 91 pT/√Hz with a 2 kHz measurement bandwidth in a magnetically unshielded environment [27].

As noted above, both NV-diamond and SiC divacancy magnetometry currently require intimate sensor-to-tissue contact, and non-invasive scalp-level deployment remains to be demonstrated for either platform. Their sensitivity ranges differ substantially as follows: NV-diamond ensemble configurations have achieved pT-level sensitivity under optimised conditions, with the most recent demonstration reaching 9.4 pT/√Hz at 1 mm standoff [56], while SiC divacancy sensors currently operate in the nanotesla-to-microtesla range, with 13 nT/√Hz reported theoretically and 3.9 µT/√Hz demonstrated in a fibre-coupled configuration [25,57]. Both remain several orders of magnitude from the femtotesla sensitivity required for scalp-level neuromagnetic recording at clinical standoff distances. The principal significance of SiC for this review is that it represents a technically credible next-generation solid-state quantum sensing platform that may eventually extend the capabilities demonstrated by NV-diamond while overcoming the green-light excitation constraint that limits biological applicability. Nevertheless, NV centres retain a more mature sensing ecosystem, including higher ODMR contrast, more extensively optimised readout methods, and a substantially larger body of biological validation [25,58]. Similarly, the sensitivity of SiC divacancy magnetometers currently remains below that of state-of-the-art NV-diamond ensemble magnetometers [59]. The clinical readiness of these modalities is summarised in Table 1.

### 3.3. Physical Constraints in Epilepsy Sensing: Standoff Distance, Sensitivity, and Bandwidth

From an engineering perspective, epilepsy sensing is constrained by three fundamental factors, the most important being the trade-off between sensor sensitivity and sensor-to-source distance (standoff). For compact current-dipole approximations, the magnetic field decreases approximately with the cube of distance in the near field, so reducing standoff is often more impactful than incremental improvements in sensor sensitivity alone. In MEG, bringing sensors closer to the cortex increases signal amplitude, improves spatial information density, and enhances source localisation accuracy [61].

Standoff also determines spatial resolution because neuromagnetic fields become progressively weaker and more spatially diffuse as they propagate through the head. By the time cortical signals reach the scalp surface, they typically lie in the femtotesla range (~10–100 fT) and are further degraded by spatial mixing arising from the heterogeneous conductivity structure of the brain, cerebrospinal fluid, skull, and scalp [62].

As a result, practical non-invasive epilepsy sensing remains confined to sensor technologies capable of femtotesla sensitivity at scalp distances, notably SQUIDs and, more recently, OPMs. Although solid-state quantum sensors such as NV-diamond and SiC divacancy magnetometers continue to improve rapidly, their current performance remains insufficient for routine scalp-level MEG without substantial reductions in standoff distance or additional signal enhancement strategies, such as gradiometric noise suppression [63]. The sensor and device dimensions, system-level infrastructure requirements, standoff characteristics, and primary use cases of each modality are summarised in Table 2.

The following four physical considerations govern the trade-offs between these modalities and their suitability for different diagnostic objectives:

(1) Standoff distance fundamentally determines the scale at which neuromagnetic signals can be measured. Sensors operating at scalp distance, including SQUID-MEG, alkali-vapour OPM-MEG, and ^4^He-OPM-MEG, record the aggregate activity of large neuronal populations and typically support source localisation at the centimetre scale, sufficient for presurgical mapping and epileptic network characterisation [15,17]. In contrast, solid-state quantum sensors such as NV-diamond and SiC divacancy magnetometers can achieve micrometre-scale spatial resolution only when biological tissue is positioned in direct proximity to the sensing element, restricting current applications largely to ex vivo preparations and microscopy platforms [24,25]. This creates a persistent trade-off between field-of-view and spatial resolution: systems capable of whole-brain coverage operate at centimetre scales, whereas systems capable of cellular resolution require near-contact sensing. No existing technology simultaneously provides whole-brain coverage and single-neuron sensitivity in vivo.
(2) Reducing sensor-to-brain distance increases measured signal amplitude and spatial information content. The transition from conventional cryogenic SQUID systems, where sensors are typically separated from the scalp by approximately 20 mm, to on-scalp OPM systems with stand-off distances of a few millimetres yields substantial gains in signal strength and source separability, providing a principal motivation for OPM-MEG development [61]. However, proximity introduces new engineering challenges. Near-scalp sensors are more susceptible to motion-induced magnetic artefacts, environmental field fluctuations, muscle activity, vascular pulsations, and other physiological interference sources. Consequently, improvements obtained through geometric proximity must be accompanied by advances in shielding, field nulling, motion compensation, and signal-processing pipelines. These limitations arise primarily from the recording environment rather than from intrinsic sensor sensitivity.
(3) Sensor bandwidth, dynamic range, and sensitivity jointly determine which epileptiform biomarkers can be measured. Alkali-vapour OPMs operating in the SERF regime achieve femtotesla sensitivity and are well suited to recording interictal epileptiform discharges and ictal activity, which predominantly occupy low-frequency bands. However, SERF operation imposes constraints on dynamic range and bandwidth. By contrast, ^4^He-OPMs trade sensitivity for substantially broader bandwidth and higher dynamic range, extending measurement capability into the kilohertz range and enabling detection of high-frequency oscillations (HFOs), which have emerged as promising biomarkers of epileptogenic tissue [42]. Sensor selection therefore depends not solely on sensitivity but on which physiological features are most relevant to the clinical question being addressed.
(4) Quantum-enhanced sensing techniques, including spin squeezing and entanglement-assisted metrology, offer a route to sensitivities beyond the standard quantum limit [67,68,69]. Nevertheless, the practical performance of clinical MEG systems is often constrained by factors external to the sensor itself, including environmental interference, patient motion, physiological background activity, and attenuation of signals originating from deep sources. Improvements in sensor noise floor alone do not mitigate these limitations. Moreover, quantum enhancement does not alter the geometric dependence of signal strength on source-to-sensor distance. Consequently, the advantages of OPM-MEG relative to SQUID-MEG derive not only from sensitivity, but also from reduced stand-off distance, wearable within a magnetically shielded environment, elimination of cryogenic infrastructure, and improved adaptability to diverse patient populations, particularly children and individuals undergoing prolonged recordings [19,37,38,39].

Taken together, current neuromagnetic sensing technologies occupy different regions of a multidimensional design space defined by stand-off distance, sensitivity, bandwidth, dynamic range, spatial coverage, and deployability. No single sensor architecture simultaneously optimises all these dimensions. Clinical utility therefore depends on selecting the sensing modality whose operating characteristics best match the diagnostic objective.

## 4. Discussion

Quantum sensing technologies do not replace the existing epilepsy diagnostic pathway—they extend it at specific points where current modalities are constrained. The following paragraphs map where OPM-MEG is currently applicable, where it is not indicated, and what developments will determine its longer-term clinical trajectory.

### 4.1. Where Quantum Sensing Is Currently Indicated

OPM-MEG is best viewed as an emerging complementary modality to SQUID-MEG in clinical contexts where MEG is already appropriate—primarily presurgical evaluation of drug-resistant focal epilepsy. We interpret the principal advantage of OPM-MEG over SQUID-MEG as geometric rather than sensitivity-based: although SQUID sensors have a lower intrinsic noise floor (3–5 fT/√Hz for magnetometer channels, versus 7–15 fT/√Hz for alkali OPMs, both expressed as magnetometer-referred field noise densities), the reduced sensor-to-brain distance achievable with on-scalp OPMs more than compensates, driving the observed improvements in signal amplitude, SNR, and source localisation. This interpretation is directly supported by on-scalp sensor-array modelling, which shows that proximity gains in signal amplitude and information content outweigh the noise-floor disadvantage of current OPMs [61], and by paediatric comparisons in which OPM-MEG achieved 2.3–4.6× higher IED amplitudes than concurrent SQUID-MEG despite its higher intrinsic noise [21]. The advantage is most pronounced in three scenarios that conventional MEG handles poorly. First, paediatric patients in whom the fixed adult-sized SQUID helmet creates a systematic size mismatch, reducing signal amplitude and limiting diagnostic yield [21,35]. Second, mesial temporal lobe epilepsy, where face-mounted OPM sensors reduce standoff distance below what any fixed-helmet system can achieve, extending coverage to anterior and deep temporal sources that represent the largest surgical candidate group but historically the lowest SQUID-MEG yield [26,60]. Third, the movement-tolerant advantage of OPM-MEG extends beyond epilepsy to other neurological conditions in which conventional MEG is limited by the requirement for stillness—a recent proof-of-principle study demonstrated feasibility in multiple sclerosis patients, in whom naturalistic posture during recording substantially improved data quality [70]. The current clinical evidence base is summarised in Table 3.

### 4.2. Where Quantum Sensing Is Not Currently Indicated

Ambulatory convulsive seizure detection is an established domain of non-EEG wearables, not quantum sensing. OPM-MEG offers no advantage for this indication—convulsive seizure detection is a peripheral signal problem addressed by accelerometery and electrodermal activity sensing. Similarly, focal and non-convulsive seizure detection in ambulatory settings remains unresolved by any current technology.

### 4.3. What Is Needed for Clinical Translation

As described in Section 3.2.3, the SERF regime requires the ambient field at the sensor to be actively nulled, and it is this field control that enables movement-tolerant recording. Advancing this capability further, real-time harmonic field modelling more than doubles the proportion of valid data trials during head movement [20,38], and lightweight MSR designs with window-coil active shielding are lowering cost and improving deployability [64]. Progress toward fully ambulatory, room-free MEG remains an active engineering challenge; reconfigurable matrix coil systems represent one promising pathway [71], but no clinically validated system outside a shielded enclosure currently exists.

OPM-MEG currently lacks the standardised reporting framework that has enabled evidence synthesis in wearable seizure detection [30]. A consensus standard specifying sensor configuration, shielding conditions, IED detection methodology, and localisation metrics referenced to intracranial or surgical outcome ground truth is a prerequisite for guideline-level evidence. The next critical step is a prospective simultaneous OPM-MEG and SEEG study with surgical outcome as the primary endpoint—a design that is now feasible given demonstrated SEEG-concordant localisation in individual cases [26,60] and the portability advantage that facilitates multicentre recruitment.

Access to MEG, and by extension to any near-term OPM-MEG clinical service, is shaped as much by reimbursement and infrastructure as by technical performance and varies markedly by region. In the United States, magnetic source imaging received FDA clearance for epilepsy use and was assigned Current Procedural Terminology (CPT) codes for epilepsy localisation and presurgical mapping in 2003, with Centres for Medicare and Medicaid Services recognition as a clinical service; reimbursement is nonetheless restricted to two indications—presurgical evaluation in drug-resistant epilepsy and eloquent-cortex mapping [72]. In several European and Asian health systems, MEG remains concentrated in a small number of tertiary academic centres and is not part of the standard presurgical pathway in the majority of institutions [36,73]. Access can be severely constrained even in high-income countries: as of 2023, only a single clinical MEG system was operational in South Korea, and Canadian economic evaluations, despite high initial capital costs, have concluded that MEG is cost-effective over the long term through improved quality-adjusted life years [74]. This uneven landscape is directly relevant to OPM-MEG, whose lower capital cost, absence of cryogenic infrastructure, and reduced siting requirements could improve access in settings where conventional SQUID-MEG is economically or logistically unfeasible—potentially its most significant health-system advantage. The regulatory landscape has also begun to move for OPM-MEG specifically. An OPM-MEG system (PyraMag Epoch, QuanMag Healthcare, Beijing, China) obtained medical-device registration in China in 2024, making it an OPM-MEG platform to receive national regulatory authorisation for clinical use, and OPM-MEG acquisitions from the same national context have been reported in large validation samples and in presurgical evaluation of drug-resistant epilepsy [75,76]. This development is a meaningful step in the transition of OPM-MEG from research use towards clinical implementation, because it establishes that an OPM-MEG system can satisfy a national regulator’s requirements for safety, manufacturing quality, and technical performance. Regulatory authorisation should nevertheless be distinguished from evidence of clinical effectiveness. Device registration in most jurisdictions, including the 2003 United States clearance of magnetic source imaging discussed above, is granted on the basis of safety and technical or substantial-equivalence criteria rather than demonstrated improvement in diagnostic accuracy or patient outcome. Market authorisation therefore removes a regulatory barrier to clinical deployment and enables reimbursement pathways to be pursued, but it does not substitute for the prospective, outcome-anchored evidence described above, nor for the standardised reporting framework that guideline-level recommendations would require. The following two requirements are complementary: registration determines whether an OPM-MEG service may be offered, whereas prospective multicentre studies with intracranial or surgical-outcome endpoints determine when it should be.

### 4.4. Integrated Monitoring and Artificial Intelligence

A practical near-term model separates continuous wearable surveillance, characterising seizure burden and multi-day periodicity [77,78], from selective MEG deployment for presurgical localisation. Cycle-informed scheduling, in which wearable-derived seizure periodicity guides the timing of MEG acquisitions to high-IED phases, is a hypothesis-generating proposal rather than a validated workflow, but could in principle improve diagnostic yield without requiring new hardware [77,78]. AI-driven automated IED detection has achieved AUC 0.987 in single-centre validation [79] and could accelerate standardisation by normalising across hardware configurations, though prospective multicentre validation against clinical endpoints remains required before deployment [7,30]. A further frontier is the convergence of quantum sensing with quantum computing and quantum machine learning: quantum algorithms could in principle accelerate MEG source localisation and automated IED classification as sensor arrays scale toward hundreds of channels, though neither application has been demonstrated in a clinical neuroimaging context [80].

### 4.5. Longer-Term Platforms

Beyond OPM-MEG, the longer-term trajectory of quantum sensing in epilepsy will be shaped by advances in solid-state and biological platforms. NV-diamond magnetometry and SiC divacancy qubits remain at the proof-of-concept stage for biological sensing, primarily constrained by the standoff problem described above. However, the development of bioinert near-infrared SiC platforms compatible with implantable chip-scale technology [25] and the demonstration of optogenetics-quantum diamond sensing synergy in mammalian tissue [48] suggest a plausible longer-term role in high-resolution near-field sensing, potentially including implantable configurations for patients already undergoing intracranial electrode placement. A conceptually distinct trajectory is offered by genetically encodable magneto-sensitive fluorescent proteins—most recently MagLOV, which demonstrates optically detected magnetic resonance in living cells at room temperature via the radical-pair mechanism [81]. Current sensitivity remains far below the femtotesla threshold required for neuromagnetic recording, but the engineerability of protein-based quantum sensors through directed evolution represents a longer-term avenue that warrants monitoring as the field develops.

### 4.6. Limitations of This Review

Several limitations should be considered when interpreting this review. First, it is a narrative rather than systematic review; no PRISMA protocol was applied, the number of records screened was not formally tracked, and literature screening and selection were performed primarily by a single author, introducing a potential for selection bias without an independent cross-checking step. Second, no formal risk-of-bias assessment was undertaken for the individual clinical studies discussed, which is a recognised limitation given the small sample sizes across the cited literature (n = 1 to n = 46). Third, the clinical evidence base for OPM-MEG derives predominantly from small, single-centre cohorts with sequential rather than randomised comparison designs, and two included sources were preprints not yet peer-reviewed at the time of writing. Finally, the quantum sensing field is advancing rapidly, and the assessments presented here reflect the evidence available as of the search date.

## 5. Conclusions

Diagnosis in epilepsy currently operates across two distinct domains. Wearable non-EEG systems have reached relative clinical maturity for convulsive seizure detection, supported by regulatory clearances and prospective multicentre data. Presurgical source localisation of epileptiform activity, from which the epileptogenic zone is inferred, remains anchored to EEG- and MEG-based workflows, with stereoelectroencephalography (SEEG) as the reference standard when non-invasive findings are insufficient.

Within this framework, OPM-MEG represents the most clinically developed quantum sensing modality applicable to epilepsy. Cohort-level studies have established technical feasibility and signal-level comparability with SQUID-MEG, with higher signal amplitudes and greater sensor placement flexibility enabling paediatric-adapted recording, ictal acquisition during natural movement, and extended coverage of deep temporal regions. The transition from technical non-inferiority to demonstrated clinical benefit—improved electrode targeting, fewer inconclusive invasive evaluations, and better patient outcomes—represents the critical next step that prospective multicentre studies must address. This assessment applies to IED detection and source localisation of epileptiform activity; no quantum sensing evidence currently addresses seizure classification, which was included in this review only to frame the wider diagnostic context.

Nitrogen-vacancy diamond and silicon carbide divacancy magnetometry remain at the preclinical stage, several orders of magnitude from the sensitivity required for non-invasive scalp-level recording, though rapid progress in solid-state sensor engineering warrants continued attention. Overall, quantum sensing does not replace the existing diagnostic pathway but extends it at specific points where current modalities are constrained. These conclusions should be interpreted in light of the narrative review design, single-reviewer study selection, absence of a formal risk-of-bias assessment, and the small, predominantly single-centre clinical evidence base.

## Figures and Tables

**Table 1:** Clinical characteristics and readiness of quantum sensing modalities for epilepsy.

Clinical Dimension	SQUID-MEG	Alkali OPM-MEG	^4^He-OPM-MEG	NV-Diamond	SiC Divacancy
**Human epilepsy evidence**	Established; large series (1000+ patients) [14]	Small cohorts (n = 5–46) [21,22,23]	Small case series (n = 1–4) [40,41]	Not demonstrated	Not demonstrated
**IED detection**	Established [13,14]	Demonstrated [21,22,23]	Demonstrated [40,41]	Not demonstrated	Not demonstrated
**Ictal recording**	Established; limited by movement intolerance	Demonstrated [22]	Not established	Not demonstrated	Not demonstrated
**Source localisation**	Established [13,14]	Demonstrated [21,22,23]	Emerging [40,41]	Not demonstrated	Not demonstrated
**Intracranial/surgical-outcome validation**	Established; SEEG and surgical outcomes [13,14]	Limited; SEEG concordance in case series [60]	Limited; concurrent SEEG comparator [40,41]	Not demonstrated	Not demonstrated
**Paediatric evidence**	Established; limited by fixed helmet geometry [21,35]	Early clinical evidence [21]	None	Not demonstrated	Not demonstrated
**HFO-band recording**	Possible within limited bandwidth; not routine	Limited by SERF bandwidth (1–100 Hz)	Bandwidth supports (DC–2 kHz); not clinically validated [41,42]	Not demonstrated	Not demonstrated
**Clinical readiness**	Established; routine at select tertiary centres [13,14]	Early clinical feasibility	Early clinical feasibility	Preclinical	Preclinical

**Table 2:** Sensor scale and standoff characteristics across quantum sensing modalities.

Sensor	Sensor/Device Dimensions	System Footprint and Infrastructure Requirements	Sensors per Helmet	Operating Environment	Intrinsic Sensitivity (Noise Floor)	Typical Sensor–Source Distance	Use Case
**SQUID-MEG (System Level)** 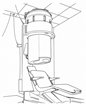	Millimetre-scale: the SQUID pickup coil and Joseph-son junction element are millimetre-scale but must be housed within the cryostat.	Massive: multi-tonne magnetically shielded room and a fixed cryogenic dewar with continuous liquid-helium supply; dedicated siting and a shielded suite are required, and the sensor array is not accessible outside the cryostat.	200–300 sensors in a standard clinical whole-head array.	Requires shielding	3–5 fT/√Hz (C)	~20 mm (further in children)	Macro-scale (~1–3 cm^2^): whole-brain, non-invasive presurgical network mapping.
**Alkali OPM-MEG (System Level)** 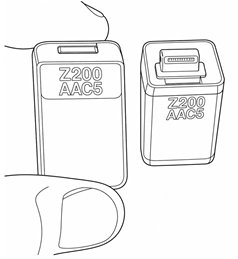	Centimetre-scale: individual sensors are approximately 2 × 2 × 5 cm.	Room-scale but cryogen-free: a magnetically shielded room with active field-nulling coils is required; lightweight MSR and window-coil designs are reducing siting cost and complexity [64].	~50 to 400+ sensors. Wearable arrays range from custom local grids to dense whole-head systems.	Narrow dynamic ~±5 nT requires shielded room or active field cancellation; bandwidth 1–100 Hz.	7–15 fT/√Hz (C)	~5 mm (on scalp)	Regional/sub-centimetre: whole-head clinical MEG, deep mesial temporal sources, and paediatric tracking.
**^4^** **He-OPM-MEG (System Level)** 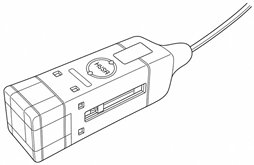	Centimetre-scale: comparable to alkali OPM sensors, slightly longer.	Room-scale but cryogen-free: a controlled magnetic environment is required, though the wider dynamic range relaxes field-nulling demands relative to SERF alkali OPMs.	~5 to a few dozen sensors. Currently validated using small, targeted local arrays.	Wide >200 nT, more tolerant but requires controlled magnetic environments; bandwidth DC–2 kHz.	40–50 fT/√Hz (E)	~5 mm (on scalp)	Regional/sub-centimetre: capturing high-frequency oscillations (HFOs) and fast ripples.
**NV-Diamond (Device Level)** 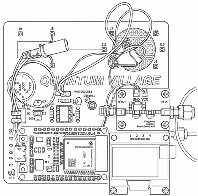	Micro- to meso-scale: atomic defect housed in a micrometre-to-millimetre-scale diamond slab or probe.	Not defined for clinical use: bench-top laser excitation, optics, and photodetection surround the diamond probe; ambient, unshielded operation is feasible in principle, but no whole-head system exists.	Typically, 1 sensor (a single NV defect or an ensemble of NV defects within one probe) under a microscope.	High dynamic range allows ambient/unshielded applications.	~100 pT/√Hz (typical ensemble); 9.4 pT/√Hz (best reported, ~1 mm standoff [56]) (E)	~10 µm	Micro-scale (nanometre to µm): detection of action potential magnetic fields from excised invertebrate neurons [24]; recent advances demonstrate sub-10 pT/√Hz sensitivity at ~1 mm standoff under ambient conditions, representing progress toward but not yet achieving scalp-level neuromagnetic recording [56]; NV-based quantum NMR also demonstrated for single-molecule and nanoscale chemical sensing [45,65,66].
**SiC Divacancy (Device Level)** 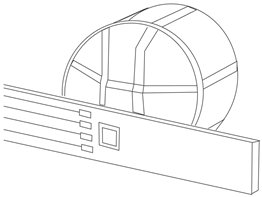	Nano- to chip-scale: atomic defects placed on an ultra-thin optical fibre tip or wafer-scale chip.	Not defined for clinical use: bench-top optical excitation and readout; fibre-coupled and wafer-scale integration demonstrated only in proof-of-concept setups.	Typically, 1 to a few sensors in highly localised, proof-of-concept experimental setups.	Ambient operation possible in principle.	13 nT/√Hz (T); 3.9 µT/√Hz fibre-coupled (E)	~1–10 nm	Nano-scale (sub-nanometre): potential near-field biological sensing; quantum NMR/nanoscale spectroscopy; implantable concepts remain speculative.

Note. For SQUID-MEG and OPM-MEG, values describe complete whole-head recording systems; for NV-diamond and SiC divacancy, values describe the sensing element only, as system-level infrastructure for clinical neuromagnetic recording has not yet been defined. Sensitivity values are labelled theoretical (T), experimental (E), or clinical (C) according to the strongest available evidence. All sensitivity values are magnetometer-referred field noise densities in field units per √Hz, quoted for magnetometer channels or as the magnetometer-equivalent noise of gradiometer channels; gradiometer noise expressed as a field-gradient density (fT/cm/√Hz) is a different quantity and is not directly comparable with these figures. Standoff distance and spatial resolution are inversely related: closer proximity enables higher spatial resolution but reduces field of view.

**Table 3:** Key clinical studies of OPM-MEG in epilepsy.

Study	n	Age Group	Design	OPM Type	Comparator	Key Findings	Validation Method
[20]	2	Adult	Prospective proof-of-concept case series	Alkali (Rb)	SQUID-MEG	IEDs detected with free head movement	Concurrent SQUID-MEG recording
[21]	14	Paediatric (school-age)	Prospective comparative cohort	Alkali (Rb)	Concurrent SQUID-MEG	IED amplitudes 2.3–4.6× higher; SNR 27–60% greater	Concurrent SQUID-MEG; dipole source concordance
[40]	1	Adult	Single-patient case study	^4^He-OPM + Alkali (Rb)	SQUID-MEG and SEEG	IED detection comparable to SQUID; ^4^He advantage for HFO-range bandwidth	Concurrent SQUID-MEG; concurrent SEEG
[41]	4	Adult	Prospective case series	^4^He-OPM	SQUID-MEG and SEEG	IED detection comparable to SQUID; noise floor 40–50 fT/√Hz vs. 3–5 fT/√Hz for SQUID (magnetometer-referred)	Concurrent SQUID-MEG; concurrent SEEG as ground truth
[22]	5	Adult	Prospective case series	Alkali (Rb)	SQUID-MEG	Ictal and interictal IEDs detected; ictal source localisation feasible	Concurrent SQUID-MEG; clinical source localisation report
[26]	Not reported	Adult	Exploratory cohort (preprint)	Alkali (Rb)	MRI lesion location	Interictal and ictal activity detected from mesial temporal structures	MRI lesion concordance; preprint—peer review pending
[23]	46	Adult	Retrospective comparative	Alkali (Rb)	SQUID-MEG	IED detection and source localisation comparable to SQUID (n = 46); consistent SNR advantage	Concurrent SQUID-MEG; dipole source concordance; lesion MRI
[60]	11	Adult	Prospective case series	Alkali (Rb); face-OPM array	SQUID-MEG; SEEG (n = 2); surgical outcome (n = 1)	Face-OPM array detected IEDs across all TLE sub-regions; SNR equal to or exceeding SQUID; added value in anteromedial TLE	SEEG concordance (n = 2); surgical resection cavity (n = 1)

## Data Availability

No new data were created or analysed in this study. Data sharing is not applicable to this article.

## Abbreviations

The following abbreviations are used in this manuscript:

AI	artificial intelligence
AUC	area under the receiver-operating curve
DC	direct current
ECG	electrocardiography
EDA	electrodermal activity
EEG	electroencephalography
EMG	electromyography
EMU	epilepsy monitoring unit
ESI	electrical source imaging
FAR	false alarm rate
fMRI	functional magnetic resonance imaging
fT	femtotesla
GTC	generalised tonic–clonic
hdEEG	high-density electroencephalography
HFO	high-frequency oscillation
IED	interictal epileptiform discharge
ILAE	International League Against Epilepsy
MEG	magnetoencephalography
MRI	magnetic resonance imaging
MSR	magnetically shielded room
NMR	nuclear magnetic resonance
nT	nanotesla
NV	nitrogen-vacancy
ODMR	optically detected magnetic resonance
OPM	optically pumped magnetometer
OR	odds ratio
PRISMA	Preferred Reporting Items for Systematic Reviews and Meta-Analyses
pT	picotesla
QML	quantum machine learning
SEEG	stereoelectroencephalography
SERF	spin-exchange relaxation-free
SiC	silicon carbide
SNR	signal-to-noise ratio
SoC	system-on-chip
SQUID	superconducting quantum interference device
SUDEP	sudden unexpected death in epilepsy
TLE	temporal lobe epilepsy
µT	microtesla
